# Characteristics of systematic lymph node dissection and influencing factors of sentinel lymph node biopsy using carbon nanoparticles in endometrial carcinoma: a single-center study

**DOI:** 10.1186/s12957-023-02922-0

**Published:** 2023-02-07

**Authors:** Siqi Tao, Zhibang Zhang, Liling Li, Xiaorui Yuan, Hongliang Chen, Yongjing Zhang, Chun Fu

**Affiliations:** Department of Obstetrics and Gynecology, The Second Xiangya Hospital, Central South University, No. 139 Ren Min Road, Changsha, 410011 Hunan China

**Keywords:** Endometrial carcinoma, Lymph node metastasis, Sentinel lymph node mapping, Prediction model

## Abstract

**Background:**

Carbon nanoparticles (CNPs) are a new tracer for lymph node mapping, which can quickly reach and develop lymph nodes through a lymphatic network. This research investigated the characteristics of systematic lymph node dissection and sentinel lymph node biopsy mapped with CNPs in endometrial carcinoma.

**Methods:**

We first applied CNPs to systematic lymph node dissection in 18 endometrial carcinoma patients as the study group and another 18 endometrial carcinoma patients who were not injected with anything served as the control group. Then, we applied CNPs to sentinel lymph nodes biopsy in 54 endometrial carcinoma patients. All 54 patients received systematic lymph node dissection after sentinel lymph node biopsy. The detection rate, sensitivity, specificity, and accuracy of systematic lymph node dissection and sentinel lymph node biopsy by CNPs were respectively analyzed. A nomogram model for predicting the success of sentinel lymph node mapping was established.

**Results:**

The average number of lymph nodes removed in the CNP-labeled study group was higher than that in the control group (*p*<0.001). CNPs improved the number of lymph nodes with a diameter ≤ 0.5cm. The detection rate, sensitivity, specificity, and accuracy of sentinel lymph nodes biopsy by CNPs for endometrial carcinoma were 70.4%, 100%, 100%, and 100%, respectively. The nomogram model included factors of long menopause time, cervical cyst, and hard cervical texture, and the area of ROC curve was 0.816.

**Conclusions:**

CNPs improve the detection rate of small lymph nodes. CNPs can trace sentinel lymph nodes in evaluating lymph node metastasis in endometrial carcinoma.

## Background

Endometrial cancer (EC) is a common malignant tumor and seriously threatens women’s health [1]. Systematic lymph node dissection (LND) is used to assess lymph node status, judge tumor stage, and guide postoperative adjuvant therapy for endometrial cancer [2]. However, systematic LND may increase the incidence of vascular injury, secondary cellulitis, and lymphedema [3]. On the other hand, the accuracy of sentinel lymph nodes biopsy (SLNB) in detecting the sentinel node and the absence of survival benefit of systematic lymphadenectomy make it possible to substitute SLNB for systematic LND [4, 5]. Sentinel lymph node (SLN) is defined as the first lymph node to receive primary tumor drainage, and it is the most likely lymph node to be metastasized in the process of lymphatic spread.

SLN mapping methods mainly include dye tracer, nuclide tracer, and combined tracer [6]. Methylene blue as a tracer is easy to operate, but has disadvantages such as short imagination time and rapid entry into the lymph node of the next station, affecting SLN recognition [6]. The detection rate of the nuclide tracer is higher than dye tracer, but it requires special equipment and has the risk of exposure to radioactive substances [6]. Indocyanine green (ICG) has the advantages of strong tissue penetration, high detection rate, and rapid development speed. However, the application of ICG is limited due to the need of a special fluorescent laparoscope to identify SLN [7]. The present situation suggests that a new SLN mapping technique with simple operation, convenient application, and high detection rate is urgently needed.

CNPs as a new lymphoid tracer may be used for SLN mapping. After the operating instruments are inserted into the abdomen, the assistant should vertically inject 1-ml CNPs into the cervix at 3 and 9 o’clock respectively [8]. The diameter of CNP is only 150nm, while the size of the lymphatic gap is 120–500nm. When injected into local tissues, CNPs can be swallowed by lymphatic system and enter lymphatic tissues through the incomplete basal membrane of lymphatic capillary wall [9]. The lymphatic vessels and lymph nodes are mapped rapidly and the staining lasts a long time. After 10–15 min of injection, the surgeon can identify the lymphatic vessels and lymph nodes dyed black by the naked eyes, and these earliest stained lymph nodes are sentinel lymph nodes. Therefore, CNPs have the characteristics of safety, efficiency, and fast staining as a tracer for SLNB [9].

SLNB with CNPs was initially used in breast cancer, colon cancer, gastric cancer, and thyroid cancer [10–13]. It was gradually applied in early-stage cervical cancer and endometrial cancer. Preliminary studies suggested that SLNB with CNPs was feasible in endometrial cancer, and cervix injection had a higher detection rate and accuracy than fundus injection [14]. The detection rate of SLNB with cervix injection of CNPs may be correlated with a variety of factors, including clinicopathological features of patients and local anatomical features of the cervix. However, influencing factors of SLNB with CNPs and related mechanisms were still unclear.

The study intended to investigate the characteristics of systematic LND and SLNB mapped with CNPs in EC. By analyzing the detection rate, accuracy, and influencing factors, we established a model to predict the success rate of SLN mapping with CNPs, which provided a basis for the application of SLNB mapped by CNPs in EC.

## Materials and methods

### Selection of patients

The research was approved by the Ethics Committee of the Second Xiangya Hospital of Central South University. At the Department of Gynecology, Second Xiangya Hospital, Central South University, 36 patients with EC were consecutively enrolled to explore the application of CNPs in systematic LND from November 2016 to April 2019. Eighteen patients were included in the study group and injected with CNPs as a tracer, while the other 18 patients were included in the control group and not injected with anything. Fifty-four patients with EC were collected to identify the use of CNPs in SLNB in our institution from March 2019 to April 2021. All of them were injected with CNPs.

The inclusion criteria were as follows: pathologically diagnosed as EC preoperatively; imaging assessed as tumor confined to the uterus; no surgical contraindications; no history of severe allergies, especially to any component of CNPs; and no preoperative radiotherapy, chemotherapy, hormone, or other treatments. The exclusion criteria were combination of other types of tumors and patient undergoing laparotomy. All patients signed informed consent forms.

### CNP injection

0.5ml of CNPs (trade name: Kanalin; 0.5ml:25mg; Lai Mei Pharmaceutical Co, Chongqing, China) was diluted with 1.5-ml normal saline, and then, we used two 1-ml syringes to extract it for cervical injection.

**Fig. 1:**
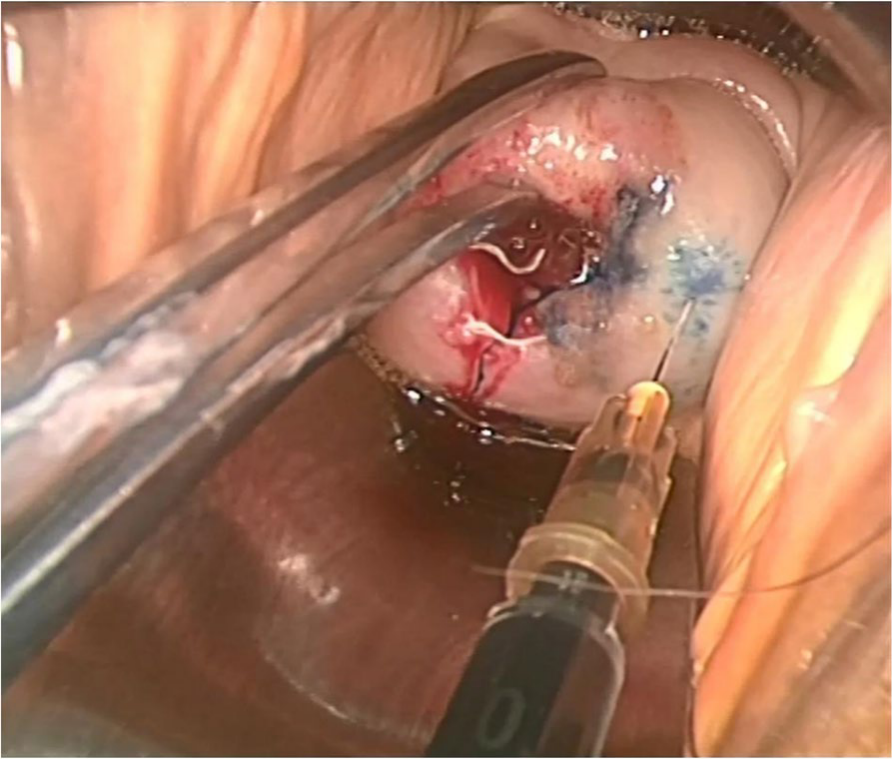
Carbon nanoparticles injected into the cervix at 3 and 9 o’clock

**Fig. 2:**
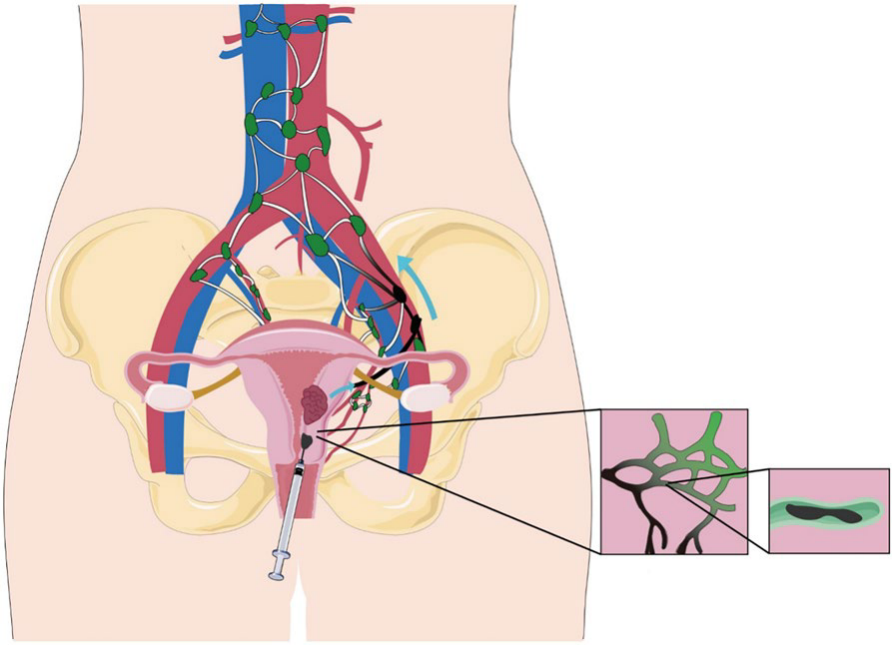
Diagram of tracer reaching lymphatic network. Deep injection into the cervical stroma can effectively inject the tracer into the lymphatic network at the beginning of the uterine body, and then, the tracer enters SLNs through the uterine lymphatic network

After the operating instruments were inserted into the abdomen, the assistant injected 1-ml CNPs into the cervix at 3 and 9 o’clock respectively [8] (Fig. 1). We adopted a combination of superficial injection and deep injection. Superficial injection ensured successful staining, while deep injection could successfully send the tracer into deep tissue. Then, the tracer could reach the lymphatic network and SLNs [4] (Fig. 2).

The needle was vertically injected into the serosal layer of the cervix for superficial injection. And after the tracer dispersed, deep injection with a depth of 1–2cm was performed. We pumped it back to avoid entering the blood vessels before each injection. The whole injection process lasted 3 min. After the injection, we gently pressed the injection points with cotton swabs to prevent leakage.

### Systematic LND and pathological examination

When applied to systematic LND, we determined that the best time to collect developed lymph nodes was 20–30 min according to our previous studies. Most lymph nodes that can be developed would be identified during this time. We carefully examined the completely exposed pelvic cavity to search for black lymph nodes and lymphatic vessels. The lymph nodes identified by CNPs or not were resected. Patients in both the study group and the control group underwent laparoscopic pelvic lymphadenectomy and para-aortic lymphadenectomy.

**Fig. 3:**
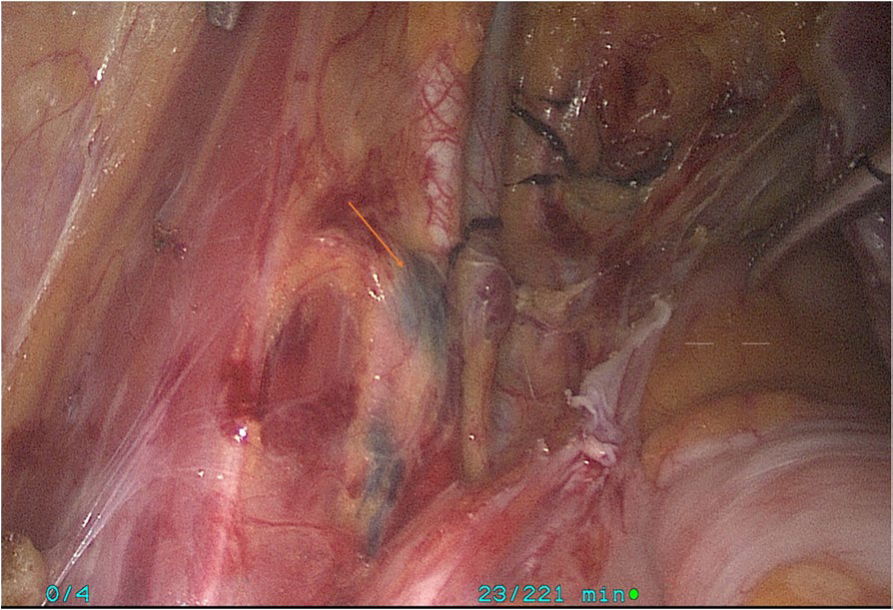
Schematic diagram of sentinel lymph node. Sentinel lymph node mapping was performed in patients with endometrial carcinoma. Carbon nanoparticles spread along the para-uterine lymphatic vessels, and the first black external iliac lymph node was found along the lymphatic vessels, which was identified as sentinel lymph node

When applied to SLNB, we could observe the passage of lymphatic vessels with black staining under laparoscope after the assistant completed the tracer injection on one side, and the earliest stained lymph node was SLN (Fig. 3). It was stipulated that the time from the beginning of injection to observing the first identified lymph nodes should not exceed 10–15 min [11, 15]. If the stained lymph nodes were found within this time, SLN mapping was regarded as a success; otherwise, it was deemed to be failed. All patients continued to undergo comprehensive staging surgery including pelvic and abdominal lymphadenectomy.

**Fig. 4:**
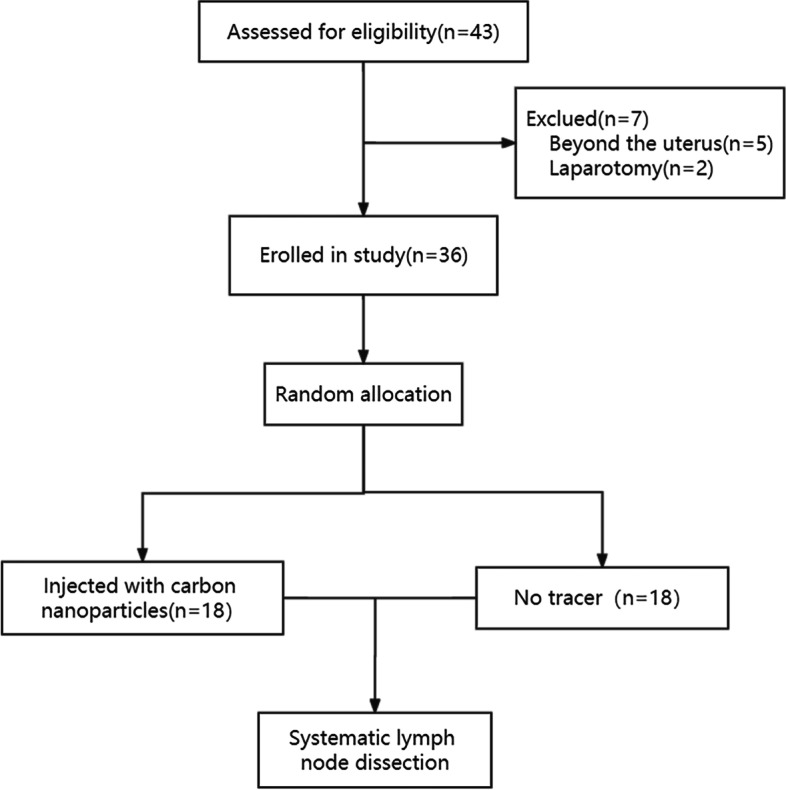
Flowchart of carbon nanoparticles applied to systematic lymph node dissection. A total of 36 patients were recruited and randomly divided into the control group and the study group. 18 patients in the study group were injected with carbon nanoparticles, while 18 patients in the control group were not injected with anything. All patients underwent systematic lymph node dissection

**Fig. 5:**
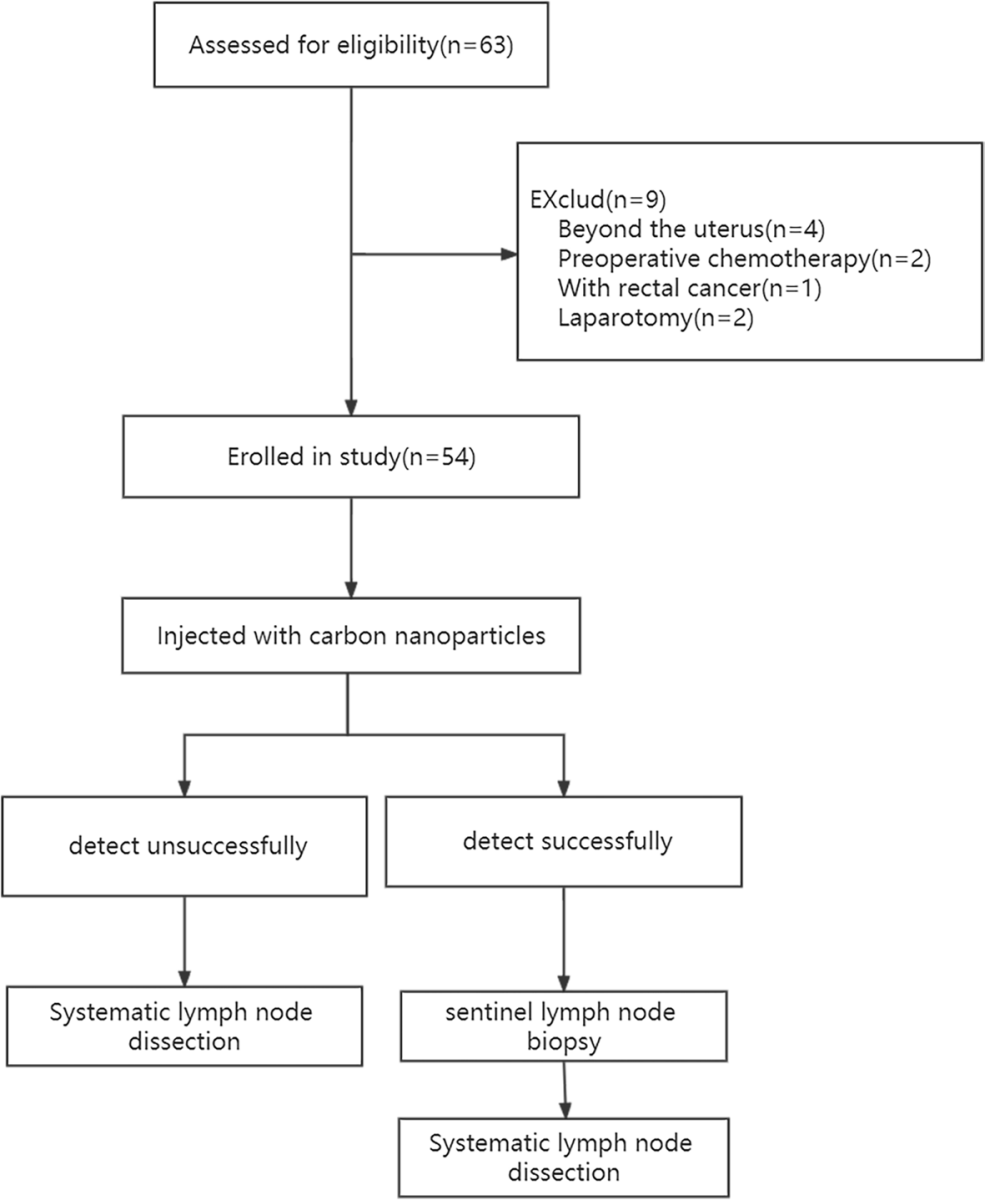
Flowchart of carbon nanoparticles applied to sentinel lymph node dissection. All 54 recruited patients were injected with carbon nanoparticles. Patients with unsuccessful imaging received systematic lymph node dissection, and those with successful imaging received systematic lymph node dissection after sentinel lymph node biopsy

The resected lymph nodes were sent to the pathology department for embedding, HE staining, routine pathological examination, and immunohistochemistry. The metastatic lymph nodes were determined by pathological examination results. We recorded locations, numbers, and pathological results of all resected lymph nodes. The flowchart is shown in Figs. 4 and 5.

### Definition of influencing factors

The cervix sizes were defined as follows: the diameter of the cervix ≤2cm was atrophy, 2 cm < cervical diameter ≤3cm is normal, and diameter > 3 cm is hypertrophy. The stiff cervix was determined by 2 experienced gynecologists after gynecological examination. And cervix locations were divided into superficial, normal, and deep by the position of the cervix relative to the position 7cm away from the hymen margin.

### Result interpretation

Pathological examination was the diagnostic standard for lymph node metastasis. When applied to SLNB, according to the evaluation standard of Louisville University [16], the calculation methods were as follows. True negativity meant no metastasis in both SLN and pelvic lymph nodes. False negativity meant that there was no metastasis in SLN, but metastases in pelvic lymph nodes. Detection rate = (number of cases with at least one developed SLN/total number of cases studied)×100%. Sensitivity = (number of cases with positive SLN/number of cases developed successfully and with pelvic lymph nodes metastases)×100%. Specificity = (number of cases with negative SLN/number of cases developed successfully and without pelvic lymph nodes metastasis)×100%. Accuracy=(number of true positive and true negative cases/number of cases developed successfully)×100%. False-negative rate=(number of cases with negative SLN but positive pelvic lymph nodes /number of cases with positive pelvic lymph nodes)×100%. Negative predictive value=(number of cases developed successfully and without metastasis /number of cases developed successfully and with negative SLN)×100%

### Statistical analysis

SPSS 26 software was used for data analyses. *χ*^2^ test was used for comparison between groups of categorical variables. Factors influencing the success of SLN mapping were analyzed using logistic analysis. A nomogram model to predict the success rate of SLN mapping was established with R language, and a ROC curve to evaluate the diagnostic efficiency of the model was drawn. *P*<0.05 meant the difference was statistically significant.

## Results

### CNPs applied in SLND

The clinical characteristics of patients enrolled are shown in Table 1. The median age of the study group and control group was 52.5 years and 50.5 years, respectively (*P*=0.19). In the study group, the numbers of patients with FIGO stage IA, II, and III were 9, 8, and 1, respectively. While in the control group, they were 8, 8, and 2 (*P*=0.24). The differences in all these clinical characteristics were not significant between the 2 groups. Patients’ vital signs were stable during the cervix injection with CNPs, and no adverse events such as allergy, fever, blood vessel injury, nerve damage, and organ impairment occurred during and after operation.

**Table 1:** Clinical characteristics of patients in study of carbon nanoparticles in systematic lymph node dissection

Parameter	Study group (*n*=18)	Control group (*n*=18)	*P*
Median age	52.5	50.5	0.19
Median BMI	23.12	22.12	0.21
Histological diagnosis			
Endometrioid adenocarcinoma	16	15	
Serous carcinoma	1	2	1.2
Clear cell carcinoma	1	1	
Depth of stromal invasion			
≤1/2	12	13	1.5
>1/2	6	5	
FIGO stage			
IA	9	8	
II	8	8	0.24
IIIC	1	2	

A total of 595 lymph nodes were removed in the study group and 447 in the control group. The average number of lymph nodes removed per patient in the study group was significantly higher than that in the control group (33.06 vs 24.83, *P* <0.001). The percentages of removed lymph nodes with a diameter ≤0.2cm in the study group and control group were 20.3% and 13.2% respectively, the percentages of removed lymph nodes with the diameter of 0.2cm–0.5cm in the study group and control group were 44.4% and 34.9% respectively, and the percentages of removed lymph nodes with the diameter of ≥0.5cm in the study group and control group were 35.3% and 51.9% respectively. The differences were significantly different (*P*=0.04) (Table 2).

**Table 2:** Composition of sizes of removed lymph nodes in two groups (*n*%)

Lymph node diameter	Study group (*n*%)	Control group (*n*%)	*P*
≤0.2cm	20.3% (121/595)	13.2% (59/447)	0.04
0.2–0.5cm	44.4% (264/595)	34.9% (156/447)	
≥0.5cm	35.3% (210/595)	51.9% (232/447)	
Total	100%	100%	

All 18 cases in the study group were successfully mapped using CNPs, and we found a total of 360 developed lymph nodes. Lymph node metastasis was found in only one case. The patient was 54 years old and in stage IIIC of well-differentiated endometrial adenocarcinoma. Four right internal iliac lymph nodes and 3 left obturator lymph nodes were found to be metastatic, and all metastatic lymph nodes were successfully identified by CNPs. The detection rate of lymph nodes was 60.5% (360/595), while the sensitivity is 100%, and the false-negative rate was 0%. No metastatic lymph node was detected in the control group.

### CNPs applied in SLNB

The median age of the 54 patients enrolled was 54.74 years, and patients with endometrioid adenocarcinoma accounted for 87% (47/54). Detailed clinicopathologic features of patients are summarized in Table 3.

**Table 3:** Clinical characteristics of patients in study of carbon nanoparticles in sentinel lymph nodes biopsy

Parameter	Cases (*n*)	Proportion (%)
Age		
≤50years	15	27.8
50–60years	28	51.9
60–70years	8	14.8
≥70years	3	5.6
Menstrual status		
Not menopause	19	35.2
Menopause ≤5 years	15	27.8
Menopause 5–10 years	9	16.7
Menopause 10–20 years	5	9.3
Menopause ≥20 years	6	11.1
Pathological types		
Endometrioid adenocarcinoma	47	87.0
Other types	7	13.0
Degree of cell differentiation		
Medium/high differentiation	44	81.5
Low differentiation	10	18.5
Tumor locations		
Cornua uteri	23	42.6
Fundus of uterus	12	22.2
Corpus uteri	14	25.9
Diffused in the uterine cavity	5	9.3
Tumor sizes		
≤2cm	35	64.8
>2cm	19	35.2
Depths of invasion		
Mucosal layer	7	13.0
≤1/2 muscularis	43	79.6
>1/2 muscularis	4	7.4
Cervical metastasis		
Yes	46	85.2
No	8	14.8
Cervical cyst		
Yes	7	13.0
No	47	87.0
Stiff cervix		
Yes	8	14.8
No	46	85.2
Cervix sizes		
Atrophy	18	33.3
Normal	30	55.6
Hypertrophy	6	11.1
Cervix locations		
Superficial	10	18.5
Normal	41	75.9
Deep	2	3.7
Out of vagina	1	1.9

Thirty-eight cases using CNPs as tracer were successfully mapped on at least one side. Twenty-two cases of them succeeded on both sides and 16 cases on only one side. The detection rate of SLN mapping with CNPs was 70.4% (38/54). There were 3 cases with lymph node metastases, and all of them were identified successfully by CNPs. One case had both SLNs and pelvic lymph node metastases, and two cases had only SLN metastases but no pelvic lymph node metastases. The sensitivity, specificity, and accuracy of SLN biopsy in EC were 100%, 100%, and 100% respectively, with a false-negative rate of 0 and negative predictive value of 100%.

**Fig. 6:**
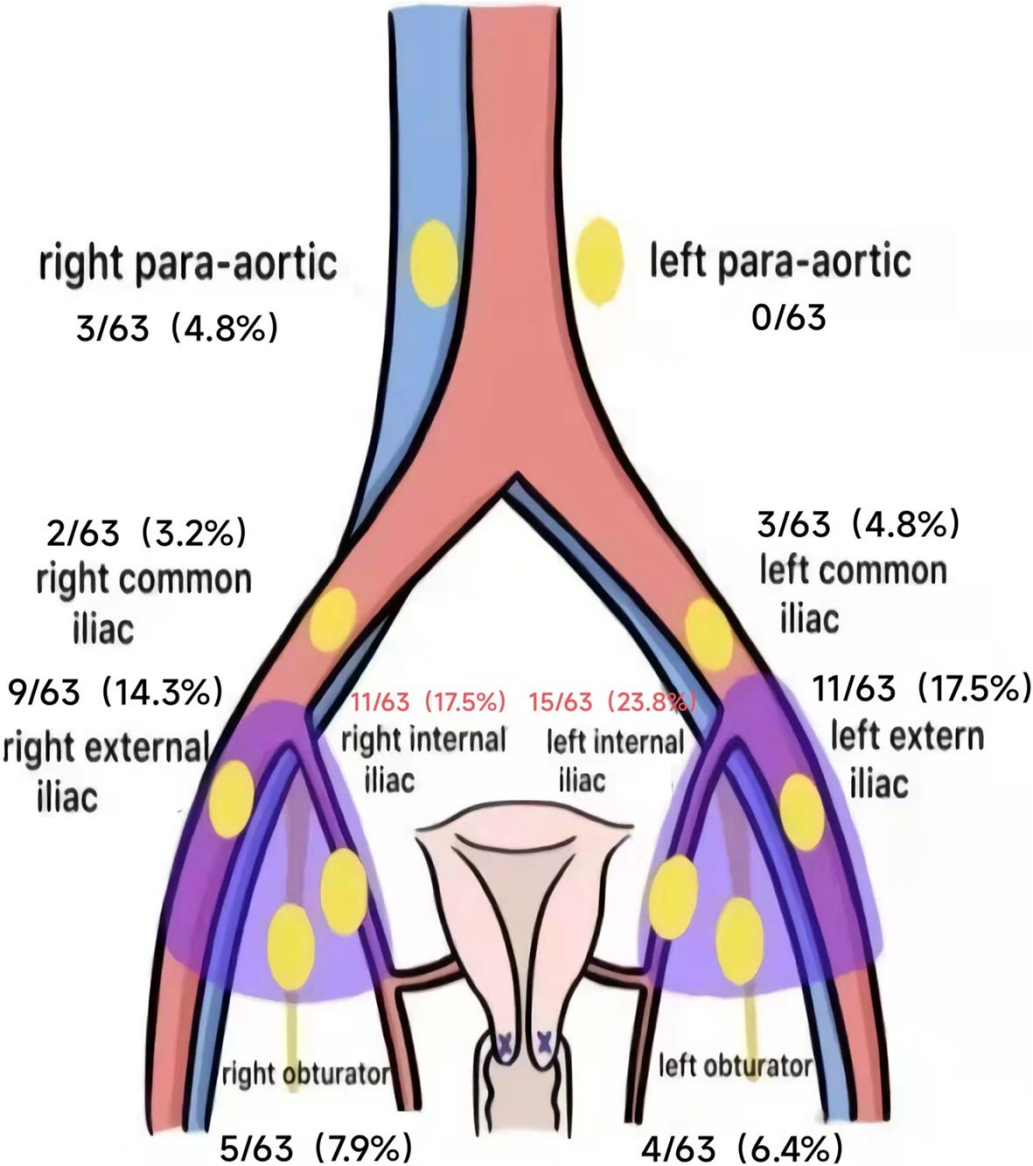
Distribution pattern of pelvic and abdominal sentinel lymph nodes

A total of 63 SLNs were detected. In patients with EC, SLNs were most commonly detected in the bilateral internal iliac regions. SLNs found in the left and right internal iliac regions accounted for 23.8% and 17.5% respectively. Distribution of SLNs is detailed in Fig. 6.

**Fig. 7:**
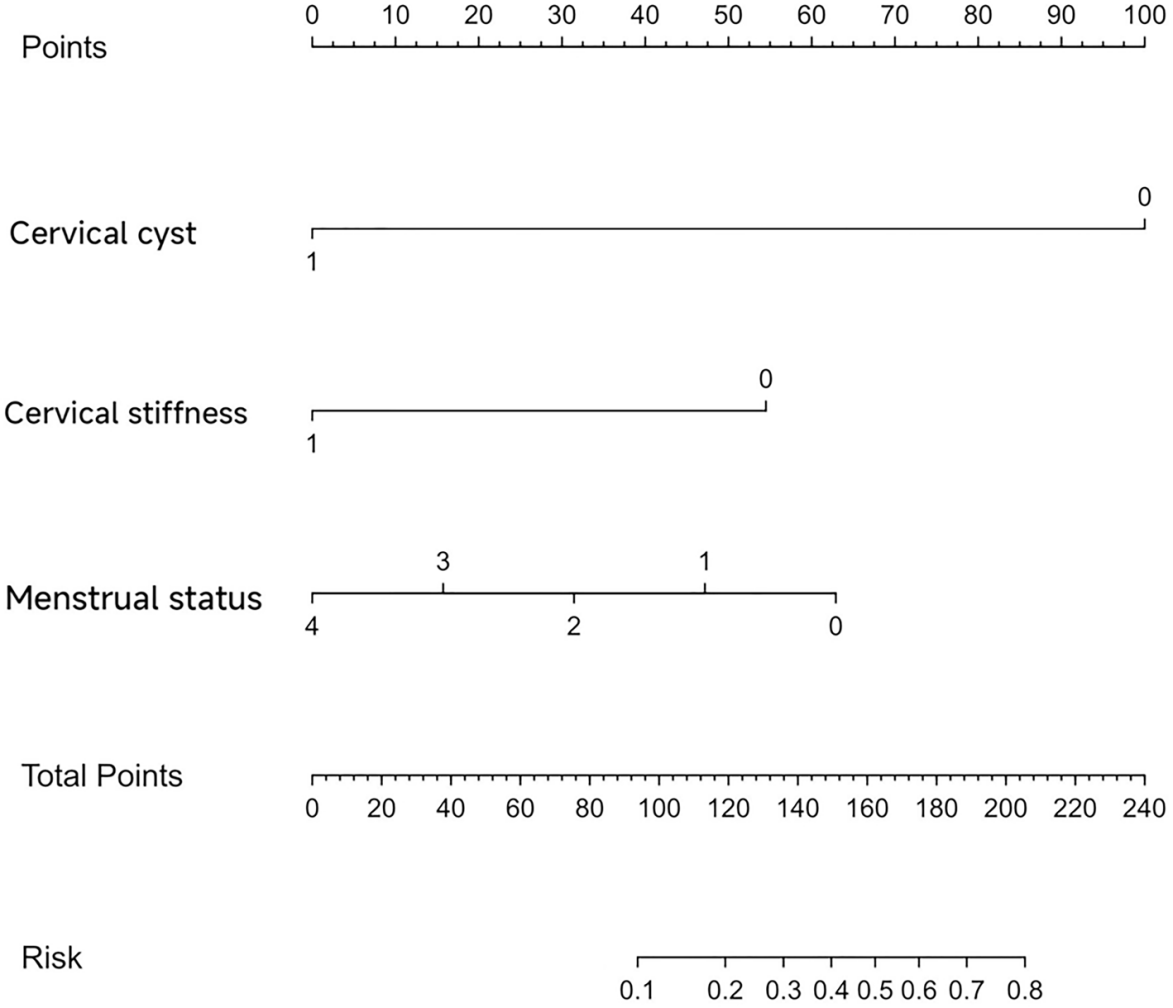
Nomogram model to predict the success rate of sentinel lymph node mapping in endometrial carcinoma

According to the analysis of patients’ clinical pathological features, we found that the detection rate of SLN mapping may be influenced by age, menstrual status, cervical cyst, cervical stiffness, and cervix sizes (*P*<0.05) (Table 4). Logistic regression analysis was carried out including these 5 factors. We determined that the long duration of menopause (*P*=0.002), cervical cyst (*P*<0.001), and stiff cervix (*P*=0.002) were risk factors for unsuccessful SLN mapping (Table 5). Including factors of cervical cyst, cervical stiffness, and menstrual status, we established a nomogram model using R language. The model could be used to assess the success rate of SLN mapping before surgery. For example, the success rate was approximately 80% of patients without cervical cyst (100) or stiff cervix (50), and before menopause (60), as shown in Fig. 7.

**Table 4:** Analysis of factors influencing detection rate of sentinel lymph nodes mapping using carbon nanoparticles

Parameter	cases(n)	SLN mapping +(*n*)	SLN mapping − (*n*)	***P***
Age				
≤50 years	15	12	3	0.006
50–60 years	28	23	5	
60–70 years	8	2	6	
≥70 years	3	1	2	
Menstrual status				
Not menopause	19	15	4	0.006
Menopause ≤5 years	15	14	1	
Menopause 5–10 years	9	6	3	
Menopause 10–20 years	5	1	4	
Menopause ≥20 years	6	2	4	
Pathological types				
Endometrioid adenocarcinoma	47	34	13	0.706
Other types	7	4	3	
Degree of cell differentiation				
Medium/high differentiation	44	34	10	0.052
Low differentiation	10	4	6	
Tumor locations				
Cornua uteri	23	17	6	0.19
Fundus of uterus	12	7	5	
Corpus uteri	14	12	2	
Diffused in the uterine cavity	5	2	3	
Tumor sizes				
≤2cm	35	24	11	0.694
>2cm	19	14	5	
Depths of invasion				
Mucosal layer	7	4	3	0.320
≤1/2 muscularis	43	30	13	
>1/2 muscularis	4	4	0	
Cervical metastasis				
Yes	46	33	13	0.913
No	8	5	3	
Stiff cervix				
Yes	8	2	6	0.009
No	46	36	10	
Cervix sizes				
Atrophy	18	8	10	0.008
Normal	30	26	4	
Hypertrophy	6	4	2	
Cervix locations				
Superficial	10	9	1	0.255
Normal	41	26	15	
Deep	2	2	0	
Out of orificium vaginae	1	1	0	
Cervical cyst				
Yes	7	0	7	<0.001
No	47	38	9

**Table 5:** Logistic analysis of factors influencing the success rate of sentinel lymph node mapping

Factors	*B*	*χ*^2^	OR(95%CI)	*P*
Cervical cyst	−26.867	3.764	0.00 (0.006–0.249)	<0.001
Cervical stiffness	−2.446	2.987	0.087 (0.005–1.388)	0.002
Cervix sizes.	−1.681	1.585	0.186 (0.008–4.163)	0.26
Menstrual status	−0.017	0.009	0.223 (0.021–2.396)	0.002
Age	0.106	2.735	1.018 (0.703–1.473)	0.098

**Fig. 8:**
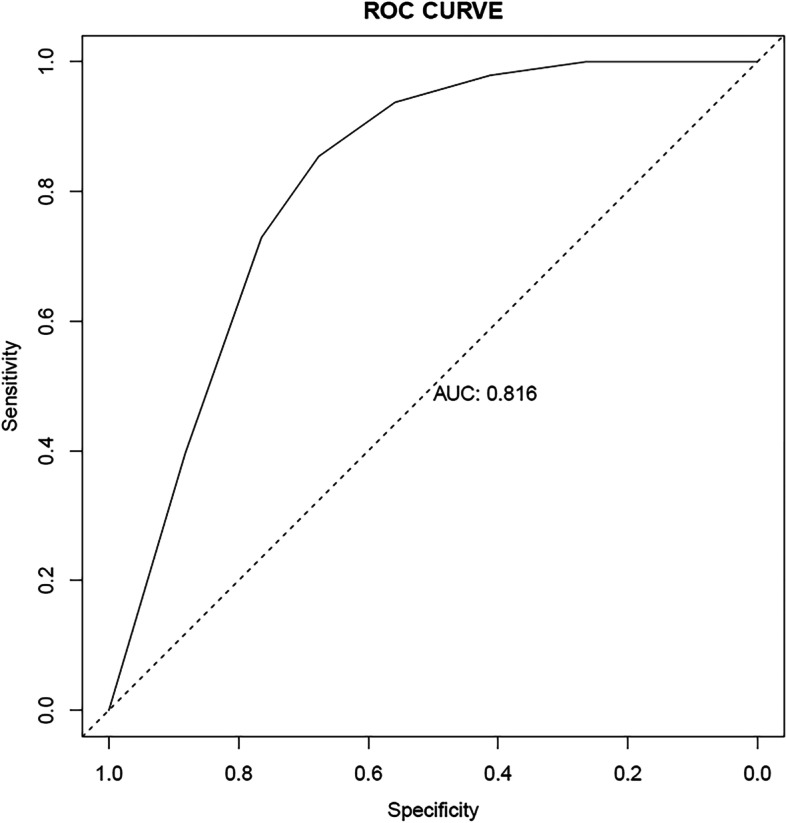
ROC curve to evaluate the discrimination of the model. The area under the curve (AUC) was 0.816

The ROC curve to evaluate the discrimination of the model was established using R language, and the area under the curve (AUC) was 0.816 (Fig. 8). As we know, the larger the AUC was, the higher the discrimination ability of the prediction model was [17]. Generally, AUC<0.6 meant the discrimination ability was weak, 0.6–0.75 meant the model had a certain discrimination ability, and > 0.75 meant the discrimination ability was great. Therefore, the model had a relatively strong discrimination ability and could better distinguish whether SLNs could be successfully developed using CNPs in patients with EC preoperatively.

## Discussion

EC is one of the most common malignant tumors of women’s reproductive system, and the incidence of EC is gradually rising. It has risen to the fourth malignant tumor in women by 2020 [18]. Nodal status obtained through systematic LND is an important part of the stage of EC [19]. However, nodal metastasis rate is relatively low in EC and systematic LND for every patient is controversial [20]. In recent years, SLNB has been proposed to access nodal status in patients with early-stage EC. Firstly, our study investigated the application of CNPs in systematic LND in EC, demonstrating the feasibility of CNPs as lymph nodes tracer. Meanwhile, we studied the application of CNPs in SLNB in EC and its influencing factors.

We found that when applying CNPs in systematic LND, the lymph node detection rate in EC was 60.5%, the sensitivity was 100%, the negative predictive value was 100%, and the false-negative rate was 0%. Besides, no adverse reaction was found in our study. It was safer than methylene blue that had a reported allergy rate of 2% [21]. In this study, we also found that CNPs could increase the number of lymph nodes removed during operation of EC, especially the small lymph nodes less than 0.5cm in diameter. The reasons are as follows. Firstly, the lymph nodes identified by CNPs will be dyed black and can be observed by surgeons with naked eyes. So we can find some tiny lymph nodes that would have been neglected, and larger lymph nodes could be easily recognized without any assistance. Secondly, the resected lymph nodes are always hidden in adipose tissue, and it is difficult to find tiny lymph nodes. When patients are injected with CNPs, tiny lymph nodes can be dyed black, which helps pathologists to identify lymph nodes that may be missed [22]. Therefore, we consider that CNPs as a lymphatic tracer can effectively detect tiny lymph nodes, providing support for the SLN micro-metastasis localization. These pieces of evidence suggest that CNPs are a suitable tracer in EC. In staging surgery of EC, whether we can substitute SLNB for systematic LND is today’s hot issue focused by gynecologic oncologists. A number of studies have shown that SLNB is feasible and effective, but requires a tracer with high sensitivity and accuracy [4, 23]. Therefore, we studied the application of CNPs in SLNB in EC. Among 54 cases of EC, 3 cases had lymph node metastases, and all of the metastatic lymph nodes were SLNs successfully mapped by CNPs. The sensitivity was 100%, false-negative rate was 0, and the negative predictive value was 100%. The results of this study showed that CNPs had a high reliability in distinguishing negative cases.

The concept of SLN was first proposed in penile tumors [24]. Studies on SLN in EC began at the end of the last century, and the detection rate was very low at the beginning of applying this technique [25]. Over the years, with the development of technique and the emergence of new tracers, the detection rate of SLN has been significantly improved. In recent years, it has been reported that the detection rate is close to 90–100% [26]. Bodurtha et al. published a meta-analysis of SLN mapping in EC, which included 55 eligible studies. The results showed that the overall detection rate of SLN mapping was 81% (95%CI: 77–84), the bilateral detection rate was 50% (95%CI: 44–56), and the detection rate of para-aortic SLN was 17% (95% CI: 11–23). The use of ICG injected in the cervix was associated with an increased detection rate (*P* < .05) [27]. At present, many scholars focus on the technique of SLNB in EC, and this technique has been written in the guidelines, indicating its feasibility. In our study, the detection rate of CNPs in 54 patients with EC was 70.37% (38/54), which was lower than that reported by other foreign researchers [28]. We considered the detection rate of SLN mapping might be influenced by age, menstrual status, tumor size, cervical cyst, and cervical texture.

There are many factors affecting the detection rate of SLN in EC, such as the type of tracers, operator’s proficiency, injection sites, tumor sites, sizes, and stages [29–32]. Taşkın et al. investigated this issue and compared the SLN detection rate of patients with different demographic, clinical, surgical, and pathological features. But they did not find these factors affected the detection rate [30]. Lucia et al. studied the prediction model of SLN mapping by cervical injection of ICG in patients with early-stage EC and mentioned that preoperative pelvic adhesiolysis and lymph node enlargement were independent risk factors for SLNB failure. Therefore, this study excluded patients with pelvic adhesiolysis and suspiciously swollen lymph nodes before surgery, but the overall detection rate of SLN mapping was still relatively low [31]. Eitan et al. introduced their studies in the algorithms of SLN mapping and examined factors that influenced the success rate of SLN mapping. In his paper, he proposed that high BMI, lymph vascular space invasion (LVSI), and surgeons with little experience were associated with SLN mapping failure. He proposed that the probability of successful SLN mapping would be more than 80% if performed by surgeons that had experience with more than 20 cases [32].

In our study, all operations were performed by the same surgeon who was specially trained. Besides, the application of CNPs in systematic LND of patients with EC was used as a preliminary experiment. Therefore, we thought that this factor cannot fully represent the cause of the failure of SLN mapping. For obese patients with high BMI, we considered that it may relate to the complete exposure of cervix. However, it was found that cervix locations did not affect the detection rate of SLN mapping in EC in our study. According to NCCN guidelines, if SLN mapping fails, systematic LND should be supplemented to ensure safety. Studies of factors affecting SLN detection rate in EC are not rare in clinical practice. However, most researchers only studied a single factor that affected the success rate of SLN mapping. And most of these studies are retrospective analyses and cannot be verified by effective intervention measures. Systematic analysis and predictive model have not been reported. Our study is the first to establish a prediction model and use the model to predict the success rate of SLN mapping in EC, providing a theoretical tool for clinical operation.

Lymph node metastasis of EC is complicated, and some scholars have classified sentinel lymphatic drainage of EC into three main approaches [14], including the upper paracervical pathway (UPP), the lower paracervical pathway (LPP), and the infundibulopelvic pathway (IPP). Among the three pathways, UPP is the most common drainage pathway that accounts for more than 90%. It is also the anatomical basis for SLN mapping by cervical injection of tracers in patients with EC. Recently, CNPs were used to confirm the existence of the three major drainage pathways. Zuo et al. observed the lymphatic drainage pathways of EC mentioned above and found that when tracers were injected into cervixes, the most common lymphatic drainage pathway was the UPP, while the IPP was only visible when CNPs were injected into the fundi of the uterus [14]. Because of this, the lymphatic metastasis of EC is quite different from that of cervical cancer. Cervical cancer passes from the primary lesion to obturator lymph nodes and internal iliac lymph nodes, then goes upward into the common iliac lymph nodes, and finally goes into the para-arterial lymph nodes [33]. However, due to the existence of the three main lymphatic drainage pathways, endometrial tumors can directly skip the lower lymph nodes and enter the upper lymph nodes, so jumping metastasis is possible for EC. In our study, SLNs were most commonly detected in the bilateral internal iliac regions, which were consistent with the UPP described above. Meanwhile, 10% of the SLNs were located in the para-aortic region, which proved the existence of the IPP. Successful detection of para-aortic lymph nodes in EC ensured the accuracy of SLNB when skip metastasis occurred. This was because we adopted a combination of superficial and deep injection. Deep injection ensured that tracers could be effectively injected to the lymph network at the beginning of the uterine body, and then entered SLNs through the lymphatic network [4].

## Conclusions

CNPs can improve the detection rate of tiny lymph nodes (≤ 0.5-cm diameter) in systematic LND of patients with EC. Meanwhile, cervix injection of CNPs can effectively trace SLNs and has a high predictive value for evaluating lymph node metastases in EC patients.

## Data Availability

The raw data of this paper are available upon reasonable request to the corresponding author.

## Abbreviations

CNPs: Carbon nanoparticles
EC: Endometrial cancer
LND: Lymph node dissection
SLNB: Sentinel lymph nodes biopsy
SLN: Sentinel lymph node
ICG: Indocyanine green
FIGO: International Federation of Gynecology and Obstetrics

## References

[1] Lu KH, Broaddus RR (2020). Endometrial cancer. N Engl J Med..

[2] Frost JA, Webster KE, Bryant A, Morrison J (2017). Lymphadenectomy for the management of endometrial cancer. Cochrane Database Syst Rev..

[3] Fotopoulou C, Kraetschell R, Dowdy S (2015). Surgical and systemic management of endometrial cancer: an international survey. Arch Gynecol Obstet..

[4] Rossi EC, Kowalski LD, Scalici J (2017). A comparison of sentinel lymph node biopsy to lymphadenectomy for endometrial cancer staging (FIRES trial): a multicentre, prospective, cohort study. Lancet Oncol..

[5] Benedetti Panici P, Basile S, Maneschi F (2008). Systematic pelvic lymphadenectomy vs. no lymphadenectomy in early-stage endometrial carcinoma: a randomized clinical trial. J Natl Cancer Inst..

[6] Staley A, Sullivan SA, Rossi EC (2017). Sentinel lymph node technique in endometrial cancer. Obstet Gynecol Surv..

[7] Papadia A, Gasparri ML, Buda A (2017). Sentinel lymph node mapping in endometrial cancer: comparison of fluorescence dye with traditional radiocolloid and blue. J Cancer Res Clin Oncol..

[8] Martinelli F, Ditto A, Bogani G (2017). Laparoscopic sentinel node mapping in endometrial cancer after hysteroscopic injection of indocyanine green. J Minim Invasive Gynecol..

[9] Vaijayanthimala V, Cheng PY, Yeh SH (2012). The long-term stability and biocompatibility of fluorescent nanodiamond as an in vivo contrast agent. Biomaterials..

[10] Sun J, Zhang J (2018). Assessment of lymph node metastasis in elderly patients with colorectal cancer by sentinel lymph node identification using carbon nanoparticles. J BUON..

[11] Wu X, Lin Q, Chen G (2015). Sentinel lymph node detection using carbon nanoparticles in patients with early breast cancer. PLoS One..

[12] Yan J, Zheng X, Liu Z (2016). A multicenter study of using carbon nanoparticles to show sentinel lymph nodes in early gastric cancer. Surg Endosc..

[13] Zhang X, Shen YP, Li JG (2019). Clinical feasibility of imaging with indocyanine green combined with carbon nanoparticles for sentinel lymph node identification in papillary thyroid microcarcinoma. Medicine (Baltimore)..

[14] Zuo J, Wu LY, Cheng M (2019). Comparison study of laparoscopic sentinel lymph node mapping in endometrial carcinoma using carbon nanoparticles and lymphatic pathway verification. J Minim Invasive Gynecol..

[15] Lu Y, Wei JY, Yao DS (2017). Application of carbon nanoparticles in laparoscopic sentinel lymph node detection in patients with early-stage cervical cancer. PLoS One..

[16] Leong SP (2011). Role of selective sentinel lymph node dissection in head and neck melanoma. J Surg Oncol..

[17] Kamarudin AN, Cox T, Kolamunnage-Dona R (2017). Time-dependent ROC curve analysis in medical research: current methods and applications. BMC Med Res Methodol..

[18] Siegel RL, Miller KD, Jemal A (2020). Cancer statistics, 2020. CA Cancer J Clin..

[19] Passarello K, Kurian S, Villanueva V (2019). Endometrial cancer: an overview of pathophysiology, management, and care. Semin Oncol Nurs..

[20] Bogani G, Murgia F, Ditto A (2019). Sentinel node mapping vs. lymphadenectomy in endometrial cancer: a systematic review and meta-analysis. Gynecol Oncol..

[21] Gien LT, Kwon JS, Carey MS (2005). Sentinel node mapping with isosulfan blue dye in endometrial cancer. J Obstet Gynaecol Can..

[22] Yu W, Cao X, Xu G (2016). Potential role for carbon nanoparticles to guide central neck dissection in patients with papillary thyroid cancer. Surgery.

[23] Persson J, Salehi S, Bollino M, Lönnerfors C, Falconer H, Geppert B (2019). Pelvic Sentinel lymph node detection in High-Risk Endometrial Cancer (SHREC-trial)-the final step towards a paradigm shift in surgical staging. Eur J Cancer..

[24] Horenblas S (2012). Sentinel lymph node biopsy in penile carcinoma. Semin Diagn Pathol..

[25] Niikura H, Okamura C, Utsunomiya H (2004). Sentinel lymph node detection in patients with endometrial cancer. Gynecol Oncol..

[26] Wang L, Liu F (2018). Meta-analysis of laparoscopy sentinel lymph node mapping in endometrial cancer. Arch Gynecol Obstet..

[27] Bodurtha Smith AJ, Fader AN, Tanner EJ (2017). Sentinel lymph node assessment in endometrial cancer: a systematic review and meta-analysis. Am J Obstet Gynecol..

[28] Holloway RW, Abu-Rustum NR, Backes FJ (2017). Sentinel lymph node mapping and staging in endometrial cancer: a Society of Gynecologic Oncology literature review with consensus recommendations. Gynecol Oncol..

[29] Capozzi VA, Valentina C, Giulio S (2021). Sentinel node mapping in endometrial cancer: tips and tricks to improve bilateral detection rate. The sentitricks study, a monocentric experience. Taiwan J Obstet Gynecol..

[30] How J, Gotlieb WH, Press JZ (2015). Comparing indocyanine green, technetium, and blue dye for sentinel lymph node mapping in endometrial cancer. Gynecol Oncol..

[31] Tortorella L, Casarin J, Multinu F (2019). Sentinel lymph node biopsy with cervical injection of indocyanine green in apparent early-stage endometrial cancer: predictors of unsuccessful mapping. Gynecol Oncol..

[32] Eitan R, Sabah G, Krissi H (2015). Robotic blue-dye sentinel lymph node detection for endometrial cancer - factors predicting successful mapping. Eur J Surg Oncol..

[33] Cea Garcia J, Rodriguez Jimenez I, Rios-Pena L (2021). Incidence and univariate models for lymphatic drainage disorders following management for cervical cancer. J Obstet Gynaecol Res..

